# Vasorelaxant effects of *Angelica decursiva* root on isolated rat aortic rings

**DOI:** 10.1186/s12906-017-1965-z

**Published:** 2017-10-02

**Authors:** Bumjung Kim, Youngki Kwon, Somin Lee, Kyungjin Lee, Inhye Ham, Ho-Young Choi

**Affiliations:** 1 Department of Herbology, College of Korean Medicine, Kyung Hee University, 26 Kyungheedae-ro, Dongdaemun-gu, Seoul, 02447 Republic of Korea; 2 Department of Herbology, Graduate School, Kyung Hee University, Seoul, Republic of Korea

**Keywords:** *Angelica decursiva*, Vasorelaxation, Hypertension, Receptor-operated calcium channels, Voltage-dependent calcium channels

## Abstract

**Background:**

Hypertension is one of the most important risk factors for cardiovascular disease (CVD) and a worldwide problem. Despite increases in the development of synthetic drugs for hypertension treatment, the rate of untreated and uncontrolled hypertension remains high. These drugs are effective, but can also cause side effects. Approximately 80% of the world population uses herbal medicines because of their low toxicity and better acceptability by the human body. Therefore, we attempted to identify natural medications for treating hypertension. The 70% ethanol extract of *Angelica decursiva* root (ADE) shows strong vasorelaxant potential, but no studies have investigated the mechanisms underlying the vasorelaxation effect of *A. decursiva*.

**Methods:**

Dried root of *A. decursiva* was identified by DNA sequencing and was extracted once with 1 L 70% ethanol (EtOH) for 3 h in a reflux apparatus at 70 °C. ADE was evaluated for its vasorelaxant effects in rat thoracic aortas. Various inhibitors of ADE-induced vasorelaxation were used.

**Results:**

ADE showed vasorelaxant effects on the intact and denuded endothelium of aortic rings pre-contracted with phenylephrine and KCl in Krebs-Henseleit solution. Tetraethylammonium and 4-aminopyridine did not alter ADE-induced vasorelaxation. However, the vasorelaxant effect of ADE was partially inhibited by pre-treatment with glibenclamide an ATP-sensitive K^+^ channel blocker. Furthermore, ADE concentration-dependently inhibited Ca^2+^ supplementation-induced vasoconstriction of aortic rings that had been pretreated with phenylephrine or KCl in Ca^2+^-free Krebs-Henseleit solution.

**Conclusions:**

These results suggest that ADE-induced vasorelaxation occurred in an endothelium-independent manner. The vasorelaxant effects of ADE were correlated with blockade of the K_ATP_ channel and inhibition of Ca^2+^ influx via receptor-operative Ca^2+^ channels or voltage-dependent Ca^2+^ channels.

## Background

Hypertension is one of the most important risk factors for cardiovascular disease (CVD) and a worldwide problem. The number of people with hypertension increased from 600 million in 1980 to 1 billion in 2008. CVD is predicted to cause one-fourth of all global deaths in 2030. Despite increases in the development of synthetic drugs for hypertension treatment, the rate of untreated and uncontrolled hypertension remains high [1]. The World Health Organization (WHO) reported that 38 million (or 68%) of 56 million human deaths worldwide were due to noncommunicable diseases (NCDs) in 2012. The four main NCDs are cardiovascular diseases (17.5 million deaths, or 46% of all NCD deaths), cancers (8.2 million, or 22%), respiratory diseases (4.0 million, or 10.7%), and diabetes (1.5 million, or 4%) [2]. Three important causes of death in Korea are cancer, cerebrovascular disease, and heart disease, causing approximately 48.3% of all deaths in Korea [3].

Hypertension is defined as blood pressure higher than 140/90 mmHg (130/80 mmHg for patients with diabetes and chronic kidney disease), whereas prehypertension is defined as a blood pressure in the range 120–139/80–89 mmHg in the Seventh Report of the Joint National Committee on Prevention, Detection, Evaluation, and Treatment of High Blood Pressure [4]. Hypertension is asymptomatic until it progresses to a life-threatening condition. If blood pressure increases continuously, the blood vessels of the heart, brain, and kidney can be damaged, leading to increased chances of renal failure, heart failure, coronary artery disease, stroke, and dementia [5]. Furthermore, if the primary conditions are accompanied by complications such as heart disease or stroke, the fatality rate markedly increases [6].

Various drugs such as diuretics, *α*-blockers, *ß*-blockers, Ca^2+^ channel blockers, angiotensin-converting enzyme inhibitors, angiotensin II receptor antagonists, and vasodilators are used to treat hypertension [7]. These drugs are effective, but also cause side effects such as dry cough [8], hyponatremia [9], impotence [10], and diabetes [11] among others. Approximately 80% of the world population uses various herbal medicines because of their low toxicity and better acceptability by the human body [12]. Additionally, one study showed that many plants used in Mexican traditional medicine for treating cardiovascular disease exhibit vasoactive and antioxidant activities [13]. Therefore, we evaluated the use of natural medications for hypertension treatment.

*Angelica decursiva* Fr. et. Sav., (=*Peucedanum decursivum* Maximowicz) is a perennial herb belonging to the Umbelliferae family of plants and grows widely in East Asia. The root of *A. decursiva* has been used in Korean traditional medicine as an expectorant, antipyretic, and cough remedy [14]. There are few studies of *A. decursiva*, which has anti-inflammatory effects [15], inhibits growth of human head and neck squamous cell carcinomas [16], and induces apoptosis of human oral cancer cells [17]. The components of essential oil from *A. decursiva* are: α-pinene (44.98%), β-barbatene (8.56%), germacrene-d (5.33%), limonene (4.21%), and β-pinene (3.81%) [18]. *A. decursiva* contains various types of coumarin derivatives such as nodakenin, nodakenetin, decursin, decursidin, umbelliferone, scopoletin, bergapten, and imperatorin [19–21].

The Umbelliferae (Apiaceae) family comprises over 300–455 genera and 3000–3750 species [22]. Numerous plants in this family have been used for both medicinal and alimentary purposes. Particularly, various herbal medicines such as *A. dahurica* [23], *Ostericum koreanum* [24], *Ligusticum jeholense* [25], *L. wallichii,* and *A. gigas* [26] were shown to have vasorelaxant effects. Thus, the purpose of this study was to screen the vasorelaxant activities of Umbelliferae medicinal plants. The 70% ethanol extract of *A. decursiva* root showed strong vasorelaxant potential; however, no studies have investigated the mechanisms underlying these effects.

## Methods

### Plant material and extraction

*A. decursiva* root was collected in Yongin, Gyeonggi province, Republic of Korea, in July 2015. The plant was identified by Professor Kyungjin Lee of Kyung Hee University. A voucher specimen (VS15071501) of the plant was deposited in the herbarium of the College of Korean Medicine, Kyung Hee University, Seoul, Republic of Korea. Dried *A. decursiva* root (100.0 g) was extracted once with 1 L 70% ethanol (EtOH) for 3 h in a reflux apparatus at 70 °C. After filtration, the extract was evaporated in a rotary vacuum evaporator (N-N series, EYELA, Japan) at 60 °C and lyophilized in a freeze-dryer (Operon™, Seoul, Korea) to obtain a brown powder (12.3 g) of crude extract. The 70% EtOH extract of *A. decursiva* (ADE) powder was accurately weighed (0.1 g), suspended in 1 ml DMSO, and placed into an ultrasonic device for 1 min for solubilization. The powder was completely dissolved, and the color was dark brown.

### Reagents and equipment

NucleoSpin Plant II kit (MACHERRY-NAGEL, GmbH & Co. KG, Germany). Blend Taq (pfu) (Toyobo, Japan). Midori green direct (Nippon genetics, Japan). NucleoSpin Gel and PCR Clean-up (MACHERRY-NAGEL, GmbH & Co. KG, Germany). ITS1 (5′-TCC GTA GGT GAA CCT GCG G-3′), ITS4 (5′-TCC TCC GCT TAT TGA TAT GC-3′), ITS2F (5′-ATG CGA TAC TTG GTG TGA AT-3′), and ITS3R (5′- GAC GCT TCT CCA GAC TAC AAT-3′) primer (Macrogen Inc. Korea). Modified Krebs-Henseleit (KH) buffer powder, Phenylephrine (PE), acetylcholine (Ach), potassium chloride (KCl), tetraethylammonium (TEA), glibenclamide, 4-aminopyridine (4-AP), calcium chloride (CaCl_2_), ethylene glycol-bis (2-aminoethylether)-*N*,*N*,*N*′,*N*′-tetra acetic acid (EGTA), and dimethyl sulfoxide (DMSO) were purchased from Sigma Aldrich (St. Louis, USA). All other reagents were of analytical purity.

In the present study a rotary vacuum evaporator (EYELA co., Japan), Freeze-dryer (Operon™, Seoul, Korea), TissueLyser2 (Qiazen GmbH, Germany), T100 Thermal cycler (BIORAD, USA), Gel Doc EZ imager (BIORAD, USA), Isometric force transducer (Grass instrument Co., USA), Powerlab data acquisition system (ADI instrument Co., Australia) were used.

### Sequencing of PCR-amplified DNA from *A. decursiva*

The dried root of *A. decursiva* was prepared for DNA sequencing. After crashing the plant sample through TissueLyser2 (Qiazen GmbH, Germany), DNA was extracted by NucleoSpin Plant II kit. For the 25 μl PCR reaction, as universal primers for PCR of the ITS region, forward primer ‘ITS1’ and reverse primer ‘ITS4’ were used. Especially, forward primer ITS2F and reverse primer ITS3R were used for ITS2 region, respectively. The PCR reaction mix contained 20 ng template DNA 1 *μ*l and 10 pmole primer 1 *μ*l in 3 *μ*l of Blend Taq (pfu). PCR conditions were as follows: 35 cycles of pre-denaturation at 94 °C for 2 min; denaturation at 94 °C for 30 s; annealing at 55 °C for 40 s and extension at 72 °C for 1 min; and final extension at 72 °C for 1 min per 1 kb. After PCR products were mixed with the Midori green direct, they separated on agarose gel for 40 min and visualized under UV light by using GelDoc. The amplified DNA band was purified with NucleoSpin Gel and PCR Clean-up kit and DNA sequence was analyzed at Macrogen Inc. (Korea).

### Animals and preparation of rat aortic rings

Forty four male Sprague–Dawley rats (240–260 g; Raonbio, Gyeonggi province, Korea) were maintained under standard laboratory conditions (22 ± 2 °C; lighting, 07:00–19:00) and were given ad libitum access to food and water. All procedures followed according to the animal welfare guidelines and were approved [KHUASP(SE)-15–066] by the Kyung Hee University Institutional Animal Care and Use Committee. The protocol of isolation and preparation of rat thoracic aorta for this study has been described previously [25].

### Experimental protocols

#### Vasorelaxant effects of ADE on PE (or KCl)-induced contraction

The aortic rings with endothelium were pre-contracted with PE (1 μM) or KCl (60 mM) in standard KH buffer. After equilibration period, various cumulative doses of ADE (25–800 μg/ml) were investigated. The vasorelaxant effect of ADE was expressed as percentages of the relaxation in response to PE or KCl.

#### Vasorelaxant effects of ADE with and without endothelium

We investigated the concentration-dependent vasorelaxant effect of ADE (25–800 μg/ml) on aorta rings with and without endothelium by PE (1 μM) or KCl (60 mM) in standard KH buffer. The vasorelaxant effect of ADE was expressed as percentages of the relaxation in response to PE or KCl.

#### Vasorelaxant effects of ADE on aortic rings with endothelium pre-incubated with various K^+^ channel blockers

We tested the vasorelaxant effect of ADE (25–800 *μ*g/ml) on aortic rings with endothelium, that were pre-incubated with a K^+^ channel blocker such as TEA (5 mM), glibenclamide (10 μM), or 4-AP (1 mM) for 20 min before PE (1 *μ*M) pre-contraction. The vasorelaxant effect of ADE was expressed as percentages of the relaxation in response to K^+^ channel blockers pre-treatment on the aortic rings.

#### Vasorelaxant effects of ADE on extracellular Ca^2+^-induced contraction contraction (via receptor-operative Ca^2+^ channels or voltage-dependent Ca^2+^ channels)

We tested the vasorelaxant effect of ADE (100–400 μg/ml) on receptor-operative Ca^2+^ channels (ROCCs) and voltage-dependent Ca^2+^ channels (VDCCs) by PE or KCl pre-treatment. We tested the contraction response induced by CaCl_2_ (0.3–10 mM) in the aortic rings without endothelium, that were pre-treated by PE (1 μM) or KCl (60 mM) in Ca^2+^-free KH buffer with and without ADE pre-incubation for 10 min. The contraction responses induced by CaCl_2_ were expressed as percentages in the presence and absence of ADE pre-treatment.

### Qualitative and quantitative HPLC analysis of standard materials in ADE

One gram of ADE was dissolved in 10 ml of methanol (HPLC reagent, J.T. Baker Co. Ltd., U.S.A.) and filtered through a 0.45 μm syringe filter (13 mm diameter and PVDF membrane, Advantec., Tokyo, Japan). The standard materials used for the qualitative analysis of ADE were nodakenin and decursin. The standards (1 mg) were serially diluted (25, 50, 100, and 200 μg/ml), and an HPLC chromatogram was obtained. Waters e2695 Alliance HPLC system connected with PDA Detector 2998 and Empower2 Software was used for the analysis. The chromatographic separation was achieved using a Sunfire C_18_ reversed-phase column (4.6 mm I.D. × 150 mm, 5 μm) (Waters, Milford, USA), with column oven temperature maintained at 20 °C. The mobile phase consisted of water (Solvent A) and 100% acetonitrile (Solvent B). The mobile phase flow rate was 1.0 mL/min with gradient elution. The percentage composition of Solvent B was maintained at 20% for 3 min, gradually increased to 30% for 5 min, maintained at 30% for 10 min, further increased to 50% for 1 min and maintained at 50% for 21 min. The injection volume was 10 μL, and UV absorbance was monitored at 330 nm. All solvents were degassed with a micro membrane filter (PTFE, Advantec., Tokyo, Japan). The quantity of the ADE standards was calculated as follows: the amount (mg) of standard material = the quantitative amount (mg) of standard materials × A_T_/A_S_/n (*n* = 3; A_T_ = the peak area of the test sample containing the standard; A_S_ = the peak area of the standard).

### Statistical analysis

All data were expressed as mean ± standard error of mean (SEM). Statistical comparisons were performed using Student’s *t*-test. All statistical analysis was performed using SPSS v.21.0 statistical analysis software (SPSS Inc., Chicago, IL, USA). *P* values less than 0.05 were considered statistically significant while *p*-values less than 0.01 were considered extremely significant.

## Results and discussion

### Sequencing of PCR-amplified DNA from *A. decursiva*

The dried root of *A. decursiva* was amplified by PCR reaction and DNA sequence was analyzed. BLAST search showed that the ITS region of this plant root was 99% identical to the strain of *A. decursiva* (GenBank ID: AY548220). Especially, ITS2 region also corresponded 99% identity with of *A. decursiva* (GenBank ID: KP334175). The DNA sequence of *A. decursiva* ITS2 region was as follow:

NNNNNGTNNCNCNNAGTCTTTGACGCAAGTTGCGCCCGAAGCCACTAGGC.

TGAGGGCACGCCTGCCTGGGTGTCACGCATCGTATTGCCTGCAGACCACT.

CACACCTGAGAAGTTGTGACGGTTTGGGGCGCAAATTGGCCTCCCGTACC.

TTGTCGTGCGGTTGGCGGAAAAACGAGTCTCCGGCGACGGATGTCGCGAC.

ATCGGTGGTTGTGAAAGACCCTCTTGTCTTGTCGCGCGAGTCCTCGTCAT.

CTTAGCGAGCTCCAGGACCCATAGGCAGCACACACTCTGTGCGCTTCGAC.

TGTGACCCCAGGTCAGGCGGGACTACCCGCTGAGTTTAAGCATATCAATA.

AGCGGAGGAAAAGAAACTTACAAGGATTCCCCTAGTAACGGCGAGCGAAC.

CGGGAACAGCCCAGCTTGAAAATTGGTCGGCTCTGCCTTCCGAATTGTAG.

TCTAGCAAGCGTCAGTGGCAGTACGTGGGGTAGATGTGTTCTGACGCGCC.

GGGCGGGGTGGCCTCTGCGCGAGACTAGAAAAATGAAAGTAGTTAAAGGA.

CCCCCGGCCGCCCACATTCTACCCCCCTTCGATGTAACAAAAGGTNTGCT.

TNATACAATTAAAATANACGTAACTANN.

### Vasorelaxant effects of ADE on PE (or KCl)-induced contraction

**Fig. 1:**
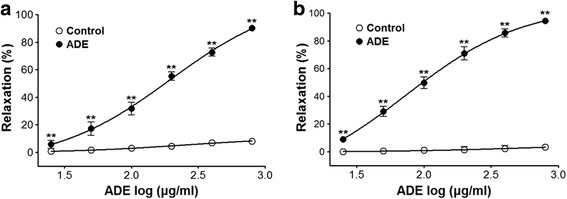
Relaxation effect of ADE (25–800 μg/mL) on PE (1 μM) (**a**) or KCl (60 mM) (**b**)-precontracted aortic rings. Values are expressed as the mean ± SEM (*n* = 5–8, number of aortic rings). ^**^ *P* < 0.01 vs. control

ADE concentration-dependently caused relaxation in PE (1 μM) or KCl (60 mM) precontracted aortic rings with intact endothelium. The maximal relaxant effect was 90.1 ± 2.0% and 94.3 ± 1.8% at the concentration of 800 μg/ml, respectively (Fig. 1).

### Relationship between vascular endothelium and ADE on PE (or KCl)-induced contraction

**Fig. 2:**
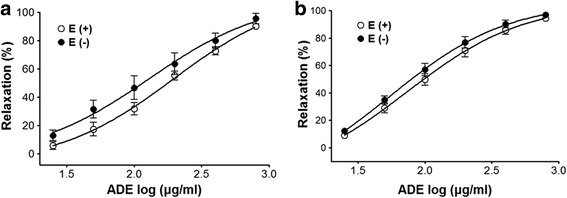
Concentration-dependent relaxant effect of ADE on PE (1 *μ*M) (**a**) or KCl (60 mM) (**b**) precontracted endothelium-intact [(E+)] and endothelium-denuded [(E-)] aortic rings. Values are expressed as mean ± SEM (*n* = 5–8, number of aortic rings)

ADE showed concentration-dependent relaxation in both endothelium-intact and endothelium-denuded aortic rings precontracted by PE (1 *μ*M) or KCl (60 mM). The maximal relaxant effect on PE-induced contraction was 90.1 ± 2.0% and 95.5 ± 3.8% for endothelium-intact and endothelium-denuded aortic rings, respectively (Fig. 2). The maximal relaxant effect on KCl-induced contraction was 94.3 ± 1.8% and 96.9 ± 2.2% for endothelium-intact and endothelium-denuded aortic rings, respectively (Fig. 2).

In this study, the maximal vasorelaxant effect of ADE was observed at a dose of 800 μg/mL. Few studies have demonstrated the various biological effects of *A. decursiva*; however, its vasorelaxant effect remains unclear. Natural products from medicinal plants have been used to treat human diseases for thousands of years. Currently, approximately 80% of the population in developing countries uses herbal medicines. The demand for medicinal plants for treating hypertension is increasing [12]. *A. decursiva* may play an important role in hypertension management in the future.

Normal endothelial cells play an important role in the human vascular system. They control blood pressure and vascular tone by secreting potent vasodilators or vasoconstrictors [27]. In the present study, ADE induced concentration-dependent relaxation, which was not related to endothelial function, in both the whole-endothelium and endothelium-removed aortic rings pre-contracted with KCl or PE, indicating that the vasorelaxant effect of ADE was endothelium-independent.

### Vasorelaxant effect of ADE on aortic rings preincubated with TEA (K_Ca_ channels blocker), glibenclamide (K_ATP_ channels blocker), or 4-AP (K_V_ channels blocker)

**Fig. 3:**
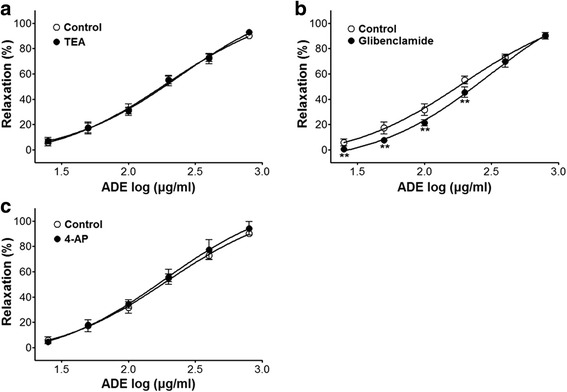
Concentration-response curves to ADE on endothelium-intact aortic rings precontracted by PE (1 *μ*M) in the presence or absence (not pre-treated control) of TEA (5 mM) (**a**), glibenclamide (10 *μ*M) (**b**), or 4-AP (1 mM) (**c**). Values are expressed as mean ± SEM (n = 5–8, number of aortic rings). ^**^ *P* < 0.01 vs. control

Incubation with TEA (5 mM) or 4-AP (1 mM) did not affect ADE-induced relaxation on endothelium-intact aortic rings contracted by PE (1 μM) treatment (Fig. 3). The vasorelaxant effects of ADE (25–200 μg/ml) on PE (1 *μ*M) precontracted endothelium-intact aortic rings were altered by glibenclamide (10 *μ*M). In the presence of glibenclamide, the ADE-induced relaxant effect was of 45.5 ± 4.2% vs. the not pre-treated control group 55.2 ± 3.1% at the concentration of 200 *μ*g/ml (Fig. 3). In these experiments, the vasorelaxant effect of ADE was not affected by endothelial function. Therefore, we concluded that there might be a correlation between the vasorelaxant effect of ADE and vascular smooth muscle.

Diverse ion channels in the vascular smooth muscle cells, endothelial cells, and pericytes play important roles in microcirculation. Microvascular smooth muscle cells have at least four different types of K^+^ channels such as inward-rectifier K^+^ (K_IR_) channels, ATP-sensitive K^+^ (K_ATP_) channels, voltage-gated K^+^ (K_V_) channels, and Ca^2+^-activated K^+^ (K_Ca_) channels [28]. To determine which types of K^+^ channels are involved in the vasorelaxant effect of ADE, various K^+^ channel blockers such as glibenclamide (K_ATP_ channel blocker), 4-AP (K_V_ channel blocker), and TEA (K_Ca_ channel blocker) were used [29]. The vasorelaxant effect of ADE was partially weakened by pre-treatment with glibenclamide. Thus, the vasorelaxant effect of ADE on rat aortic rings is correlated with K^+^ channels, particularly K_ATP_ channels.

### Effect of ADE on extracellular Ca^2+^-induced contraction (via ROCCs or VDCCs)

**Fig. 4:**
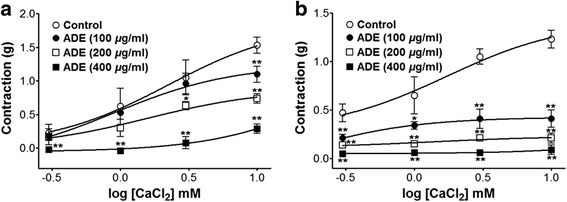
Inhibitory effect of ADE (100–400 *μ*g/ml) on the contraction induced by extracellular Ca^2+^ in endothelium-denuded rat thoracic aorta rings pretreated by PE (1 *μ*M) (**a**) or KCl (60 mM) (**b**) in the presence or absence (not pre-treated control) of ADE. Values are expressed as mean ± SEM (n = 5, number of aortic rings). ^*^ *P* < 0.05, ^**^ *P* < 0.01 vs. control

In Ca^2+^-free KH buffer, the addition of CaCl_2_ (0.3–10 mM) induced gradual increased tension on endothelium-denuded aortic rings by PE (1 μM) or KCl (60 mM) treatment. Preincubation with ADE (100–400 μg/ml) significantly inhibited the contractions induced by extracellular CaCl_2_ (10 mM) and the contraction at ADE (400 *μ*g/ml) was decreased to 0.29 ± 0.07 g and 0.09 ± 0.05 g compare to the not pre-treated control group 1.53 ± 0.12 g and 1.23 ± 0.09 g, in cells pre-contracted by PE and KCl, respectively (Fig. 4).

Vascular smooth muscle contraction is triggered by an increase in intracellular calcium, which is released from intracellular Ca^2+^ stores [30]. Extracellular Ca^2+^ influx through ROCCs or VDCCs increases intracellular calcium levels. Vasodilatation is caused by inhibition of extracellular Ca^2+^ entry through ROCCs or VDCCs in the plasma membrane [31]. ROCCs and VDCCs can be activated by PE and KCl, respectively [32]. ADE inhibited vasoconstriction induced by supplementation of Ca^2+^ in the aortic rings that had been pre-constricted with PE or KCl in Ca^2+^-free KH buffer. This finding suggests that ADE considerably inhibited the permeation of extracellular Ca^2+^ via ROCCs or VDCCs activated by PE or KCl, respectively.

Many compounds such as decursin, imperatorin, isoimperatorin, umbelliferone, bergapten, and nodakenin have been isolated from *A. decursiva*. Previous studies have shown that decursin and nodakenin have antihypertensive effects. Decursin and nodakenin lowered the blood pressure in the carotid artery of rabbits [33], and a decursin and decursinol angelate mixture showed a significant vasorelaxant activity in male Sprague–Dawley rats [34]. Similarly, various compounds including decursin or nodakenin may be responsible for the vasorelaxant effects of *A. decursiva*. Hence, *A. decursiva* is thought to be effective as a vasodilator for treating hypertension. Additional studies of these compounds may help to determine the exact mechanism underlying the vasorelaxant effect of ADE in rat aortic rings.

### Qualitative and quantitative HPLC analysis of standard materials in ADE

**Fig. 5:**
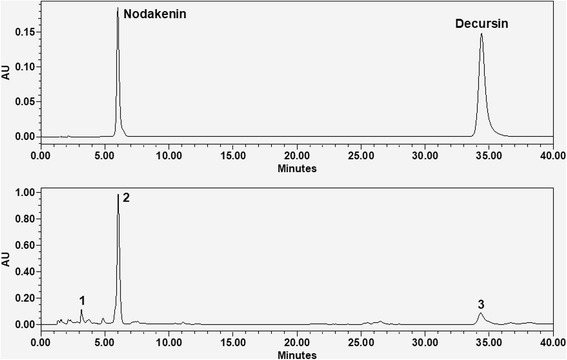
Qualitative and quantitative HPLC analysis of standard materials in ADE. The retention time of the peak 1, peak 2 (nodakenin), peak 3 (decursin) was 3.2, 6.1, and 34.4 min, respectively

The retention times of the peaks in HPLC were 3.2 min (peak 1), 6.1 min (peak 2, nodakenin), and 34.4 min (peak 3, decursin) (Fig. 5). The standard curve was calibrated by using the linear regression derived from the peak area. The regression equation (correlation coefficient, *R* ^2^) of nodakenin and decursin were *y* = 26,923.15*×* - 21,599.52 (0.999) and *y* = 55,531.57*×*-39,679.81 (0.999), exhibited good linearity. The content of nodakenin and decursin in 1 g of ADE were 0.562 ± 0.003 mg (nodakenin) and 0.063 ± 0.001 mg (decursin).

## Conclusions

In conclusion, (1) the vasorelaxant effects of ADE were endothelium independent, (2) K^+^ channels such as K_ATP_ channels were partly related to ADE-induced vasorelaxation, and (3) ADE relaxed the aortic rings by blocking the entry of extracellular Ca^2+^ via ROCCs and VDCCs.

## Abbreviations

4-AP: 4-aminopyridine
Ach: Acetylcholine
ADE: The 70% EtOH extract of *A. decursiva* root
CaCl_2_: Calcium chloride
CVD: The cardiovascular disease
DMSO: Dimethyl sulfoxide
EGTA: Ethylene glycol-bis (2-aminoethylether)-*N*,*N*,*N*′,*N*′-tetra acetic acid
K_ATP_: ATP-sensitive K^+^ channels
K_Ca_: Ca^2+^-activated K^+^ channels
KCl: Potassium chloride
KH: Krebs-Henseleit
K_IR_: Inward-rectifier K^+^ channels
K_V_: Voltage-gated K^+^ channels
NCDs: Noncommunicable diseases
PE: Phenylephrine
ROCCs: receptor-operative Ca^2+^ channels
TEA: Tetraethylammonium
VDCCs: voltage-dependent Ca^2+^ channels
